# Post-traumatic myositis ossificans of the sternocleidomastoid following fracture of the clavicle: a case report

**DOI:** 10.1186/1757-1626-1-413

**Published:** 2008-12-22

**Authors:** Shelain Patel, Andrew Richards, Ravi Trehan, Gil T Railton

**Affiliations:** 1 Department of Trauma & Orthopaedics, Kingston Hospital, Kingston-upon-Thames, Surrey, UK

## Abstract

**Background:**

Fractures of the clavicle are common injuries. The complications have been well documented in the literature.

**Case presentation:**

Despite it close proximity to the sternocleidomastoid muscle and myositis ossificans recognised as a known complication of any fracture, the two have never previously been described in association secondary to a fracture of the clavicle. We present a case where myositis ossificans affecting the sternocleiodomastoid was detected in the post-injury phase of a clavicle fracture.

**Conclusion:**

This case highlights that traumatic myositis ossificans circumscipta can arise in the sternocleidomastoid muscle following a fracture of the medial third of the clavicle.

## Background

Fractures of the clavicle are common, accounting for 7% of all fractures [1]. The complications of this fracture are well documented, but traumatic myositis ossificans circumscipta as a complication has not been previously described. We present a case of a 46 year old male whose clavicle fracture was complicated by traumatic myositis ossificans of the sternocleidomastoid muscle. Both fracture and complication were managed non-operatively.

## Case report

**Figure 1:**
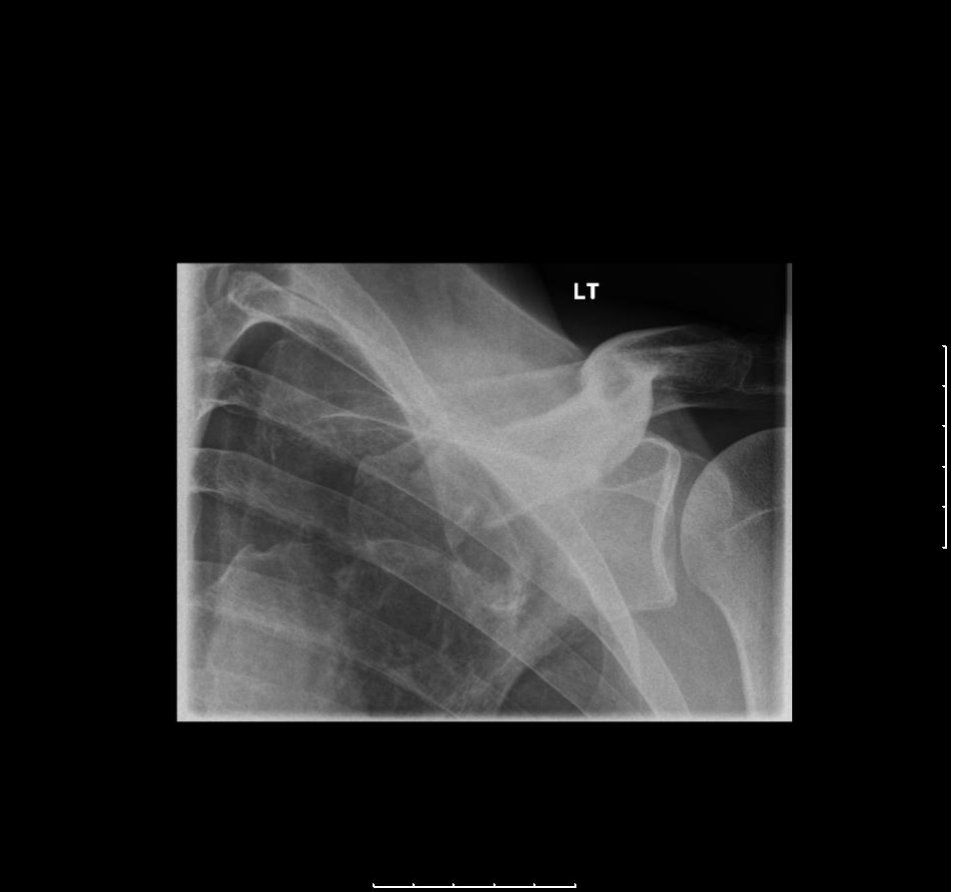
**Oblique radiograph of the left clavicle demonstrating a fracture of the medial third (day 0)**.

A 46 year old gentleman presented to the emergency department following a fall onto his left side whilst intoxicated with alcohol. His past medical history included alcohol dependency and peptic ulcer disease. He complained of pain over the medial half of the left clavicle. Examination revealed swelling and bony tenderness over the clavicle 3 cm distal to the left sternoclavicular joint. There were no documentation of masses in his neck and movement in his neck was unchanged from normal. Radiographs showed a displaced fracture of the proximal 1/3 of the left clavicle (Figure 1). He was given a broad arm sling by the emergency department and was referred to the out-patient fracture clinic for follow up.

**Figure 2:**
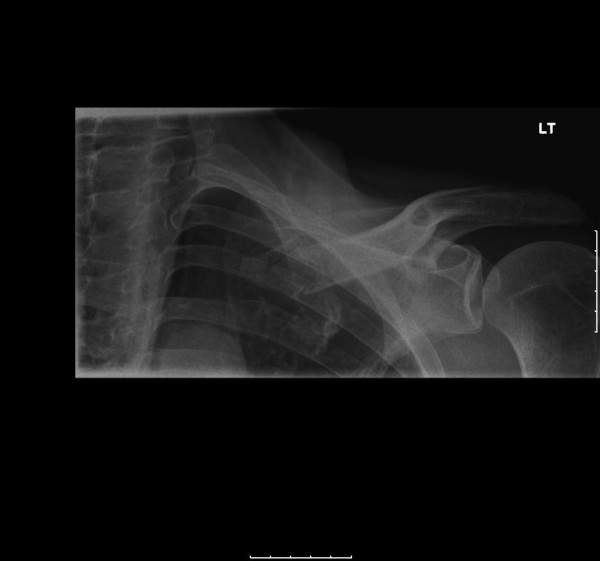
**AP radiograph of the left clavicle demonstrating a fracture of the medial third with an opacity extending from the fracture site in the soft tissues of the neck (day 8)**.

**Figure 3:**
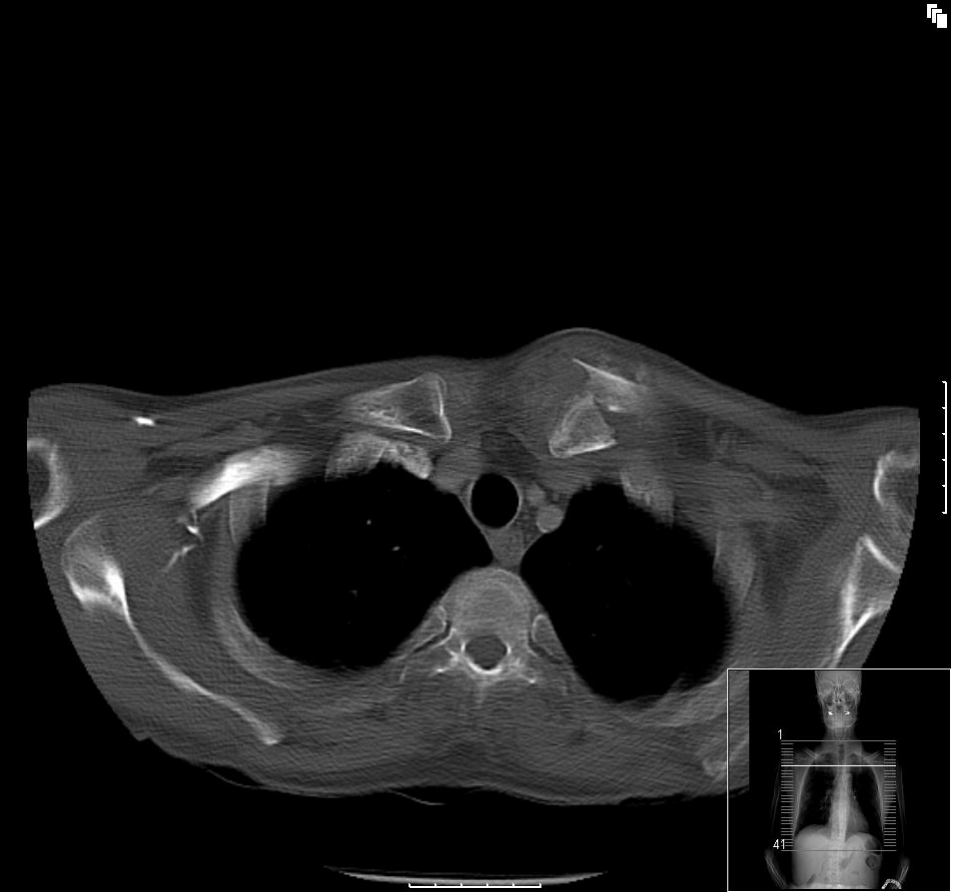
**Axial CT image showing a displaced fracture of the left clavicle (day 8)**.

**Figure 4:**
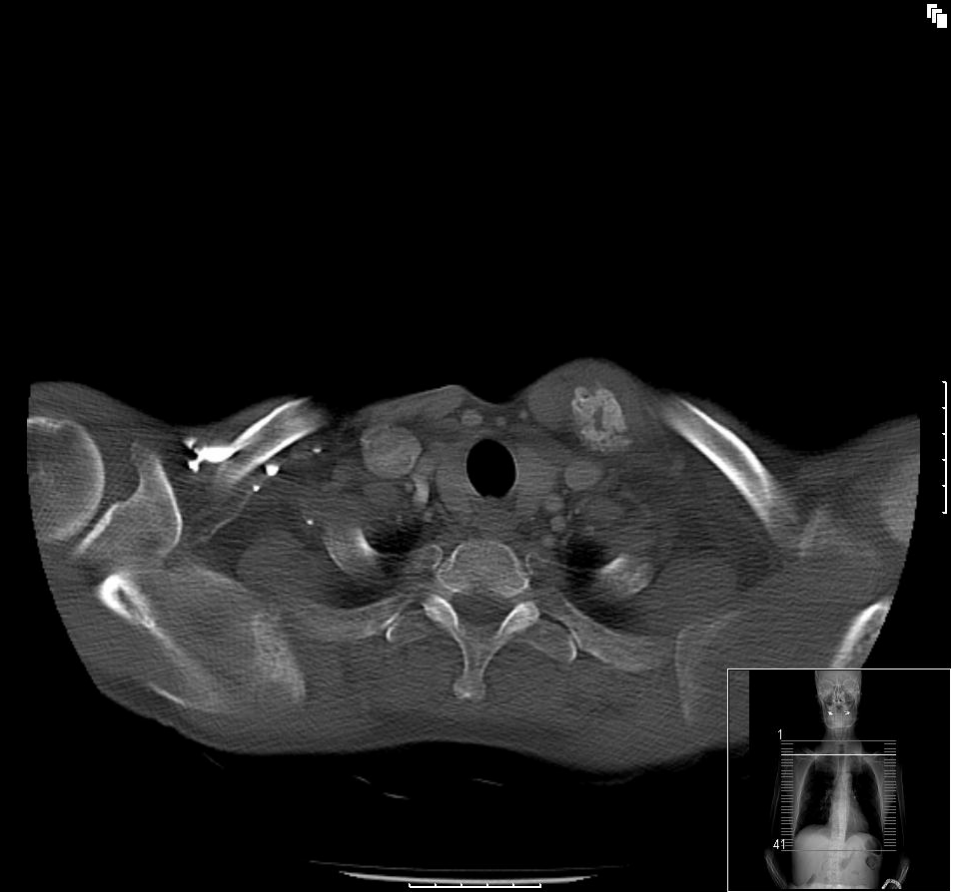
**Axial CT image showing a well circumscribed osseous lesion within the sternocleidomastoid muscle above the site of the fracture (day 8)**.

**Figure 5:**
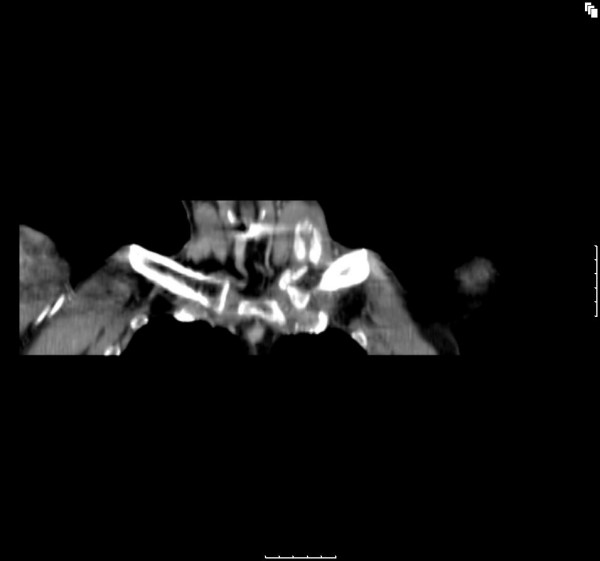
**Reformatted coronal CT image showing a fracture of the clavicle with an osseous lesion within the sternocleidomastoid muscle (day 8)**.

He was seen 8 days later in the out-patient fracture clinic. Examination revealed marked bruising over the fracture site. Furthermore, a hard, well-circumscribed mass arising from the left clavicle and extending into the sternocleidomastoid muscle was identified. Follow up radiographs were taken (figure 2). Based on the radiographic findings of extra-osseous calcification, a CT scan was performed (figures 3, 4 &5). This demonstrated an extra-osseous calcification within the sternocleidomastoid muscle.

Traumatic myositis ossificans circumscripta was diagnosed. The patient was informed that his condition would be managed non-operatively. Non-steroidal anti-inflammatory medications were not prescribed due to his past history of peptic ulcer disease and current excessive alcohol usage. Plans were made for him to be followed up in the out-patient clinic to monitor his condition, but he failed to attend any further follow-up appointments.

## Conclusion

Myositis ossificans is described as extra-osseous, localised, non-neoplastic formation of bone and cartilage [2]. It can be classified in to three types [3]:

(1) *Myositis ossýficans progressiva *which is a metabolic disorder occurring in children with widespread metamorphosis of muscle into bone, all of the skeletal muscles becoming involved progressively.

(2) *Traumatic myositis ossificans circumscripta *which follows local trauma which may be either acute or chronic repeated injuries.

(3) *Myositis ossýficans circumscripta without history of trauma*. This is usually found in paraplegia, chronic infections, burns and poliomyelitis, but may occur independently of these conditions.

The aetiology of traumatic myositis ossificans is unknown. The most common sites to be affected are the hip, anterior thigh (quadriceps), and anterior arm (brachialis) [4,5]. It appears to be a self limiting disease with spontaneous resolution after maturation in most cases, though some can take years to resolve [6].

Histologically, the "zone phenomena" has been described by Ackerman [2] where the lesion is separated in to inner, middle and outer zones.

• Central zone: extreme variation of cells and atypical mitotic figures

• Middle zone: orientated osteoid

• Outer zone: well formed bone

Myositis ossificans of the sternocleidomastoid muscle was first described in 1950 [7] though this was due to myositis ossificans progressiva, rather than following trauma. Post-traumatic cases of myositis ossificans of the sternocleidomastoid have been described, but no case has previously been reported as a consequence of a fracture of the clavicle.

This case highlights that traumatic myositis ossificans circumscipta can arise in the sternocleidomastoid muscle following a fracture of the medial third of the clavicle.

## Consent

Written informed consent was obtained from the patient for publication of this case report and any accompanying images. A copy of the written consent is available for review by the Editor-in-Chief of this journal.

## Competing interests

The authors declare that they have no competing interests.

## Authors' contributions

SP, AMR, RT and GTR were involved in the clinical care of the patient and drafting of the manuscript. All authors have read and approved the final manuscript.
